# Fibronectin Mechanobiology Regulates Tumorigenesis

**DOI:** 10.1007/s12195-015-0417-4

**Published:** 2015-08-15

**Authors:** Karin Wang, Bo Ri Seo, Claudia Fischbach, Delphine Gourdon

**Affiliations:** Department of Materials Science and Engineering, Cornell University, 327 Bard Hall, Ithaca, NY 14853 USA; Department of Biomedical Engineering, Cornell University, Ithaca, NY 14853 USA; Kavli Institute at Cornell for Nanoscale Science, Cornell University, Ithaca, NY 14853 USA

**Keywords:** Fibronectin conformational flexibility, Fibronectin mechanics, Tumor stroma, Tumor progression

## Abstract

Fibronectin (Fn) is an essential extracellular matrix (ECM) glycoprotein involved in both physiological and pathological processes. The structure–function relationship of Fn has been and is still being studied, as changes in its molecular structure are integral in regulating (or dysregulating) its biological activities *via* its cell, matrix component, and growth factor binding sites. Fn comprises three types of repeating modules; among them, FnIII modules are mechanically unstable domains that may be extended/unfolded upon cell traction and either uncover cryptic binding sites or disrupt otherwise exposed binding sites. Cells assemble Fn into a fibrillar network; its conformational flexibility implicates Fn as a critical mechanoregulator of the ECM. Fn has been shown to contribute to altered stroma remodeling during tumorigenesis. This review will discuss (i) the significance of the structure–function relationship of Fn at both the molecular and the matrix scales, (ii) the role of Fn mechanobiology in the regulation of tumorigenesis, and (iii) Fn-related advances in cancer therapy development.

## Fn and Its Significance in Cancer

**Figure 1:**
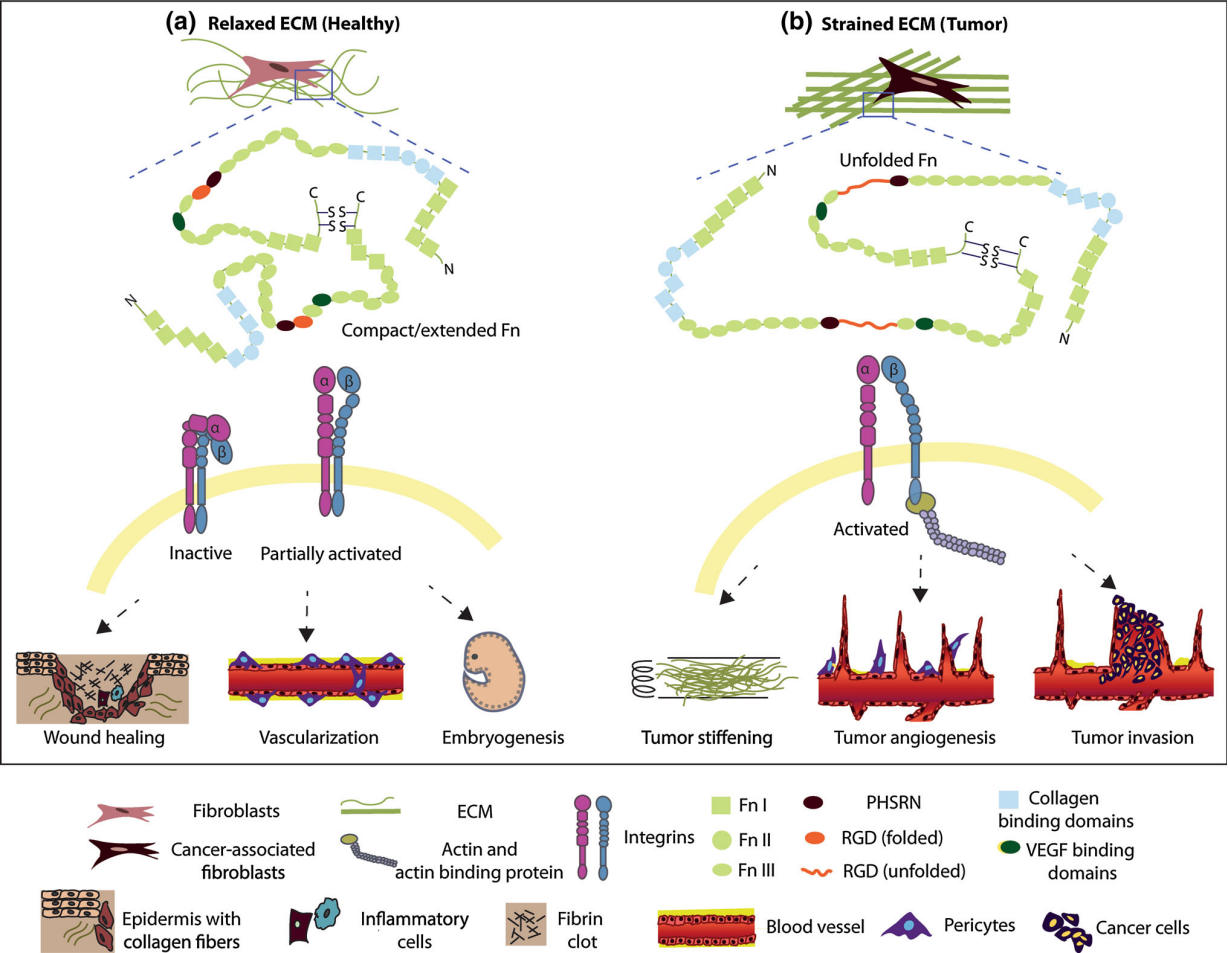
Structure-function relationship of fibronectin in healthy and pathological environments. In healthy environments, the ECM is in balance between relaxed and strained conformations to maintain normal tissue homeostasis. However, the ECM in tumor stroma has lost its integrity and starts mediating an altered ECM phenotype. The relaxed ECM (a) contains mostly compact/extended fibronectin, whereas in the strained ECM (b), fibronectin undergoes conformational changes to resist cell-mediated traction forces. These changes to fibronectin lead to exposure of soluble factor (i.e., VEGF) binding and cryptic sites. Furthermore, these conformational changes facilitate specific matrix component binding to mediate ECM remodeling or modify integrins engagement and activation (i.e., *α*v*β*3 vs. *α*5*β*1) to consequently modulate cell behaviors. Moreover, unfolded Fn is correlated with enhanced fiber strain and bulk ECM stiffening. Therefore, tumor-associated fibronectin assembled in tumor stroma facilitates a cascade of dysregulated downstream signaling for tumor progression.

Fibronectin (Fn) is one of the most abundant extracellular matrix proteins (ECM) along with collagen. Fn was first discovered as a high molecular weight fibroblast cell surface protein in the early 1970s,^64^,^126^ and then as an extracellular fibrillar network surrounding fibroblasts through immunofluorescence and scanning electron microscopy.^155^ Early isolation of Fn revealed a dimeric glycoprotein with two subunits measuring ~220 kDa^165^ held together by disulfide bonds.^66^ Most Fn is synthesized by hepatocytes to circulate in the bloodstream as soluble plasma Fn. Various cells also secrete Fn, named cellular Fn, to be directly assembled into an insoluble fibrillar network. Plasma and cellular Fn mediate different biological behaviors; plasma Fn is essential in clots during early wound healing, whereas cellular Fn mediates late wound healing, neovascularization, and angiogenesis (Fig. 1a).^50^,^147^ Fn is also implicated in other physiological (e.g., embryogenesis^156^) and pathological^30^ (e.g., fibrosis, cancer) processes.

Originally, Fn was discovered because fibroblast cells lack a cell surface protein after viral transformation.^64^,^155^ However, the loss of Fn is not a good marker of malignancy, as some anchorage-independent tumorigenic cell lines are still able to assemble a fibrillar Fn network.^73^ Further studies assessing the role of Fn in malignancy reveal high concentrations of plasma Fn after mice were inoculated with Ehrlich tumor cells,^167^ but plasma Fn fluctuates with clinical events such as chemotherapy.^24^,^168^ Other reports addressed the controversial deposition of Fn in tumors and found that it is absent in tumors but abundant in the surrounding stroma.^8^,^144^ As such, understanding Fn dynamics, i.e., Fn deposition and remodeling during tumorigenesis, is essential to expanding our knowledge of cancer.

The tumor stroma is a complex microenvironment in which components are recruited or remodeled to facilitate invasive growth and metastasis.^43^,^54^ Therefore, specific focus is placed on understanding how the surrounding ECM is altered to mediate tumor progression.^75^,^130^ Cancer-associated fibroblasts (CAFs) are major sources of increased ECM deposition and altered remodeling^97^ to create tracks for cancer cell invasion.^46^ This review will discuss (i) the importance of Fn structure, matrix assembly, and mechanics in invasive tumor growth, and (ii) their relevance to improve therapeutic strategies and diagnostic tools.

## Fn Mechanoregulation of Various Cellular Activities

Fn is a mechanoregulator of the ECM due to its conformational flexibility^14^,^35^,^38^,^159^ in both plasma^1^ and fibrillar forms.^2^,^139^ Fn consists of 3 repeating modules: FnI, FnII, and FnIII.^30^,^124^ FnI and FnII are mechanically stable modules as they are stabilized by disulfide bonds, but FnIII lack these disulfide bonds and are sensitive to external mechanical forces.^67^ FnIII modules are made up of 7 *β* strands within 2 anti-parallel *β* sheets surrounding a hydrophobic core, with FnIII_10_ holding a RGD loop (cell-binding site) between the F (6th) and G (7th) *β* strands.^96^ The RGD sequence is a ubiquitous cell binding region as it has also been found in other proteins such as fibrinogen, vitronectin, laminin, and thrombospondin.^125^ Fn contains two sites that collaboratively confer adhesion,^112^ the RGD site on FnIII_10_ and the PHSRN synergy site located on the adjacent FnIII_9_.^6^ Simultaneous engagement to both RGD and PHSRN sites is essential for integrins *α*_5_*β*_1_^111^ resulting in a binding that is highly sensitive to Fn molecular conformation.^85^ In contrast, the binding of most other integrins, including *α*_v_*β*_3_ integrins, requires engagement only to the RGD loop and is not (or less) sensitive to Fn conformation.^79^ Briefly, the RGD loop is separated from the PHSRN site by 30–40 Å and a small rotation between FnIII_9_ and FnIII_10_ orients the two cell binding sites on the same side of the Fn molecule.^88^ Therefore, any change either in the orientation (i.e., in the relative angles between the two adjacent modules) or in the spacing between adjacent modules (e.g., as it occurs during FnIII_10_ unfolding and shown in Fig. 1b), alters the type of transmembrane receptors used by cells to bind to the Fn matrix,^106^ and the subsequent downstream signaling. Another important region on Fn essential to mechanoregulation is the FnIII_12–14_ sequence, which binds various growth factors^98^ for sustained, localized signaling. Immobilization of growth factors modulates different downstream signaling.^3^ Specifically, Fn-bound vascular endothelial growth factor (VEGF) mediates structured vascularization whereas soluble VEGF directs large, leaky vasculature.^90^ Thus, Fn conformational flexibility is able to regulate cell activity *via* integrin specificity and growth factor binding.

Various cells are able to incorporate plasma Fn into the predominantly cellular Fn based-ECM of any tissue^32^,^114^ Additionally, fibroblasts are able to deposit a Fn matrix by secreting and assembling Fn into fibers at the cell periphery.^145^ Cells’ integrins *α*_5_*β*_1_,^42^*α*_3_*β*_1_,^162^ and *α*_4_*β*_1_^133^ binding to Fn were shown to participate in Fn matrix assembly. Assembly requires mechanical stimulation provided by cellular traction forces to induce a conformational change in Fn and expose cryptic binding sites that mediate Fn polymerization.^18^,^92^ Recent advances in super-resolution microscopy such as direct stochastic optical reconstruction microscopy provide insight to the ordered structure of Fn within bundled fibers, demonstrating that Fn molecules are aligned within fibers with alternating N-terminal and C-terminal overlapping regions.^45^ Fn maturation follows deposition and involves the polymerization of nascent deoxycholate-soluble Fn (12–20 nm in diameter)^22^ ultrathin fibrils into mature deoxycholate-insoluble Fn thick fibrils networks (up to 200 nm in diameter).^45^,^102^ Although multiple Fn conformations coexist in the matrix (and in individual fibrils), the average Fn conformation has been reported to evolve during ECM maturation from compact/extended Fn in early fibrils to extended/unfolded Fn in mature fibrils and matrices.^11^,^87^,^139^ The polymerization of Fn in extended conformations^47^ stimulates cell growth,^142^ a process that may be mediated by interactions with heparin sulfate proteoglycans (another matrix component to which Fn binds).^62^,^141^ Fn networks may also be initiated *via* self-assembly. Fn contains conformational-dependent^59^ binding sites for itself located on FnI_1–5_, FnIII_1–2_, FnIII_4–5_, and FnIII_12–14_.^45^ These Fn–Fn interactions may be mediated by interactions with FnIII_10_.^57^ Furthermore, fragments of these binding sites have been shown to inhibit Fn–Fn interactions and Fn fibrillogenesis.^103^,^108^ Thus, changes to initial Fn conformations are also crucial in the regulation of Fn binding to other ECM components (including itself), and modulate further ECM deposition and remodeling.

The assembly of an initial Fn network^138^ is often a prerequisite for the downstream deposition of collagen.^101^,^140^,^148^ Reciprocally, the co-deposition of collagen has several effects on the initial Fn matrix: it assists further Fn remodeling by matrix metalloproteinases such as MT1-MMP,^135^ it stabilizes the ECM,^140^ it promotes cell proliferation and maintenance of microtissue morphology (ECM reorganization),^134^ and it facilitates cell migration.^143^ The reported co-localization of both Fn and procollagen within the cell further demonstrates a likely synergistic relationship between these two ECM proteins.^89^ Fn contains a large (multimodular) collagen binding site^51^ located on modules FnI_6_FnII_1–2_FnI_7–9_.^68^ Fn regions within this site^119^ collectively bind^76^ to the collagen *α*1(I) chain between residues 757 and 791.^33^,^81^ Collagen binding stabilizes the 90° kink between FnI_6_FnII_1–2_FnI_7_ and FnI_8–9_,^36^ which is believed to assist Fn in maintaining a compact/relaxed conformation in the stroma, further regulating normal tissue homeostasis.

Fn-coated beads restrained by optical traps reveal cells’ ability to sense their environment and to respond to increased external resistance^25^ due to the strengthening of cytoskeletal tension, as later confirmed by traction force microcopy.^154^ Additionally, lysophosphatidic acid (from platelets) mediates Rho-activated stress fiber formation and enhances Fn matrix assembly, revealing the importance of cellular tension in Fn fibrillogenesis.^12^,^169^,^171^ Briefly, *α*_5_*β*_1_ integrins translocating along actin cytoskeletal bundles elongate Fn molecules^117^ with varying amounts of force,^113^ which initiates Fn polymerization and induces cytoskeletal tension.^58^ L8, an antibody known to bind Fn within FnI_9_ and FnIII_1_ and to inhibit Fn fiber assembly when added to fibroblast culture medium,^23^ increases its binding to Fn when Fn monolayers deposited on rubber substrates (cell-free system) were mechanically strained to expose a cryptic binding site.^173^ These studies suggest that isolated Fn must unfold to bind to itself and begin the fibrillogenesis process. Detailed analysis of Fn matrix assembly and maturation indicates that Fn fibers are highly elastic^65^,^82^,^115^ and heterogeneous as they comprise multiple molecular conformations, from compact/relaxed to extended/unfolded.^87^,^139^ The elasticity of Fn fibers can be attributed to the conformational flexibility of FnIII modules (lacking disulfide bonds) that are allowed to extend/unfold upon cellular traction, as suggested by steered molecular dynamics simulations^86^ and fluorescence resonance energy transfer.^139^ Importantly, an *in vivo* study also portrays the critical role of Fn conformational changes in modulating tissue function (e.g., the exposure of FnIII_1_ mediated by skeletal muscle contraction leads to vasodilation).^60^ Collectively, Fn’s cell-induced changes in conformation implicate this glycoprotein as a critical mechanotransducer in translating mechanical signals from the external environment into biochemical signals mediated by integrin clustering and cytoskeletal tension.^26^,^150^

## Roles of Conformation and Mechanics of Fn in Tumorigenesis

In fetal tissues and cancers, cellular Fn is larger^100^ and alternatively spliced^132^ to contain the following sequences: IIICS, ED-A, ED-B, which confer additional conformational changes to Fn.^10^,^16^,^39^,^149^ Fn ED-A is found at sites of tissue remodeling and during dysregulated signaling, it promotes a fibrotic phenotype^136^ for tumorigenesis^77^ and for neovascularization of metastases.^127^ This splice variant enhances VEGF-C secretion *via* the PI3 K/Akt signaling pathway.^163^ Fn ED-A secreted by endothelial cells (isolated from tumors) also induces epithelial-mesenchymal transition of cancer cells by activating the FAK-Src signaling pathway *viaα*_9_*β*_1_.^116^,^137^ Instead, Fn ED-B is found in the tumor stroma^72^ and in the tumor vasculature.^19^ This splice variant of Fn has been found to enhance cell adhesion and formation of focal adhesions for cell spreading.^56^ ECM stiffening, a hallmark of cancer, has been found to enhance ED-B splicing of Fn to propagate a tumorigenic phenotype.^13^ Thus, changes in conformation, mechanics, and alternative splicing of Fn synergistically regulate tumorigenesis.

Fn is up-regulated in the tumor stroma.^30^ Its enhanced synthesis^110^ is attributed to CAFs, fibroblasts with altered phenotype and function.^74^ CAFs are activated by TGF-*β*^83^ or transformed by Fn-tissue transglutaminase complexes contained in microvesicles released from cancer cells.^5^ Breast tumor CAFs deposit an initially dense, unfolded^20^ and stiff^153^ Fn matrix that facilitates an ‘integrin switch’, i.e., a change from primarily *α*_5_*β*_1_ binding (that depends on Fn conformation) to that of mostly *α*_v_*β*_3_ binding (that is independent of Fn conformation),^34^,^151^ resulting in enhanced pro-angiogenic (VEGF) secretion.^152^,^153^ Changes to the material properties of Fn can in turn mediate a cascade of signaling events for tumorigenesis (e.g., ECM unfolding, stiffening, tumor angiogenesis, and tumor invasion) (Fig. 1b).

Under conditions of normal tissue homeostasis, Fn mediates strong cellular adhesion. Upon matrix maturation during healthy ECM remodeling (e.g., wound healing, vascularization, embryogenesis) (Fig. 1a), Fn gradually unfolds while cells become more contractile and develop strong fibrillar adhesions containing *β*_1_ integrins.^4^*α*_5_*β*_1_ integrins binding to Fn stimulates myosin II^44^ and RhoA-GTPase to form robust peripheral fibrillar adhesions.^29^ These strong adhesive forces between Fn and *α*_5_*β*_1_ integrins (~93 pN)^94^ reduce migration of invasive cells.^69^ Fn conformational changes are often responsible for an ‘integrin switch’ as Fn conformation alters the type of integrins cells may utilize to bind to the surrounding ECM. As detailed in Section II, the most abundant Fn integrins, *α*_5_*β*_1_, require both the synergy and the RGD sites located on FnIII_9_ and FnIII_10_, respectively, to form complexes with Fn, which implies that strong *α*_5_*β*_1_-Fn binding is conformation-dependent and occurs only when Fn is in a nearly compact conformation. In contrast, *α*_v_*β*_3_ integrins require only the RGD site, i.e., weaker *α*_v_*β*_3_-Fn binding is conformation-independent and occurs even when Fn is unfolded during ECM remodeling.^28^ Weak Fn-*α*_v_*β*_3_ adhesions^131^ by cancer cells then lead to greater cytoskeletal reorganization for enhanced migration capacity^9^ and resistance against anoikis (Fig. 1b).^170^ Once Fn conformation is altered during tumorigenesis, cell–matrix interactions are dysregulated and changes to downstream signaling take place.

As Fn contains binding sites for cells, growth factors, and matrix components, variations in Fn conformation during tumorigenesis alter multiple microenvironmental interactions. The up-regulation of Fn combined with the preferred utilization of *α*_v_*β*_3_ in the tumor stroma mediates the release and activation of matrix metalloproteinase-2 (MMP-2), which favors tumor invasion and metastasis.^71^,^128^ The resulting remodeled Fn, likely degraded by MMP-2, may in turn bind with altered affinity^37^ to collagen ECM, which may lead to the formation of dysregulated, crosslinked, and stiff Col I^93^ tracks for enhanced invasion by cancer cells.^27^,^122^ Although the deposition of collagen usually requires the presence of provisional Fn, enhanced secretion of TGF-*β* does lead to collagen fibrillogenesis and fibrotic ECM remodeling even in the absence of Fn.^107^

Besides conformation, stiffness of the ECM also plays a role in tumorigenesis.^55^,^93^,^120^ ECM stiffening not only promotes Fn ED-B splicing and Fn unfolding for a pro-angiogenic integrin switch, but also contributes to TGF-*β* activity,^99^ a phenomenon that can influence myofibroblast differentiation^7^,^160^ or epithelial to mesenchymal transition (EMT) for tumor progression.^91^ Invasive cells preferentially migrate towards stiffer ECM (durotaxis).^95^,^121^ Durotaxis is mediated by both the recruitment of *α*_v_*β*_3_ integrins that re-organize and reinforce the cytoskeleton^9^,^123^ at the leading edge of cells^70^ and the extensions of filopodia.^161^ This rigidity response is attributed to activation of p130Cas *via* Fyn recruitment by receptor-like protein tyrosine phosphatase alpha (RPTP*α*) at the leading edge of these cells.^84^ As altered Fn is stiffer, it may direct cancer cell invasion into the surrounding stroma for eventual metastasis.

Finally, Fn binding to cell surfaces *via* integrins also mediates clustering of growth factor receptors.^164^ Enhanced levels of VEGF^41^ are secreted by breast cancer cells (and/or fibroblasts subjected to paracrine signaling by breast cancer cells^53^ ) for tumor angiogenesis.^31^,^48^ An isoform of VEGF, VEGF_165_, increases breast cancer and endothelial cell migration in presence of Fn (and heparin).^104^ Specifically, Fn forms a complex with VEGF-receptor-2 and *α*_5_*β*_1_^78^ to bind VEGF^157^ on the heparin II binding domain located on FnIII_13–14_.^158^ Furthermore, ECM components such as heparin or heparan sulfate facilitate an extended conformation of Fn to enhance VEGF binding^105^ in a pH-dependent manner.^49^ As acidic environments promote tumorigenesis,^118^ low pH in the tumor stroma may contribute to these changes in Fn conformation and subsequent tumor angiogenesis. Overall, the Fn matrix is not only a mechanotransducing network but also a chemical reservoir of signaling molecules for cells, as Fn-bound VEGF facilitates organized vascular sprouting and branching^90^*via* enhanced activation of MAPK through *β*_1_ mediated clustering of VEGFR2.^21^

## Development of Fn-Based Cancer Therapy

During tumorigenesis, primary structure, conformation, and mechanics of Fn are altered, which clearly affects its multiple biological functions. As the ECM stiffens, alternative splicing of Fn increases,^13^ which leads to additional conformational changes (in an already highly strained and stiff tumor-associated matrix^153^) and promotes dysregulated downstream cell–matrix interactions for tumor progression. Targeting this altered Fn during tumorigenesis is therefore extremely desirable. Using phage antibody technology, molecular probes were successfully developed to distinguish between different unfolded (strained) states of Fn.^15^ Additionally, CGS-1 and CGS-2 human antibody fragments were isolated and found to directly target Fn containing ED-B in human tissues as well as in other species.^17^ Another antibody fragment specific for ED-B, scFv(L19), was fused to domains of interleukin-12 to enhance cellular immunity, which led to slower tumor growth and reduced metastasis^52^ while injection of a radioactive-homodimer form of the fusion protein,^123^ I-L19(scFv)_2_, in cancer patients demonstrated the potential to image primary and metastatic tumors noninvasively.^129^ Using *E.* *coli* expressing bacterial thioredoxin, therapeutic vaccines specific against ED-A and ED-B were also developed and found to stimulate anti-ED-A and anti-ED-B antibodies to reduce tumor growth.^40^,^63^ Biologically active fragments of Fn were also utilized, particularly for structure–function experiments portraying the ability of Fn to bind to itself and regulate its own function.^61^,^103^ Among them, a fragment derived from the first type III repeat in Fn, FnIII_1C_ (named anastellin),^109^ was reported to inhibit tumor growth, angiogenesis, and metastasis.^166^*In vitro* experiments studying the mechanisms behind anastellin’s effects revealed that anastellin bound Fn and induced a conformational loss of a Fn epitope containing ED-A, which then activated MAPK and filopodia to stimulate Fn remodeling.^80^ However, anastellin was also shown to mediate an inflammatory and pro-angiogenic phenotype of stromal cells within tumors.^172^ Finally, Fn-derived N-terminal and C-terminal heparin-binding domains, respectively named heparin I and heparin II domains, were also found to inhibit cancer cell adhesion and invasion by reducing *α*_v_*β*_3_ expression and MMP-9 activity.^146^ As advances are made in our knowledge of Fn nanostructure (e.g., splice variants), assembly (e.g., fibrillogenesis, molecular arrangement in fibers, strain-induced conformational changes, remodeling), and mechanics (e.g., contributions of both elastic and viscoelastic properties of Fn to direct cell behavior) during tumorigenesis, the development of therapeutic strategies and diagnostic tools will continue to improve to mitigate tumorigenesis.

## Conclusions and Future Perspectives

Fn is able to trigger a wide range of cellular activities and is extremely dynamic, constantly undergoing remodeling processes where one or more of its essential properties are modified. Modulation of Fn dynamics is likely a strategy for tumor stromal cells to respond to microenvironmental changes (in particular, paracrine signaling from cancer cells) and contributes actively to tumorigenesis. However, because of the reciprocal nature of cell–Fn interactions, it is still unclear whether early Fn alterations in the tumor microenvironment are a cause or a consequence of the disease.

Numerous Fn-linked tumorigenesis mechanisms still need to be unraveled. As both plasma and cellular Fn play an inextricable role in mediating different biological functions, delineating their respective contributions during tumorigenesis must be addressed. Neither the mechanisms responsible for Fn assembly into fibers nor the detailed molecular structure of fibers are well understood, which would certainly help in defining the full range of parameters that regulate the Fn structure–function relationship. Although it is now well accepted that Fn assembly is dysregulated during tumorigenesis and leads to altered materials properties of the entire Fn network, it is likely that other microenvironmental disorders, such as altered MMP activity, additionally drive changes in Fn remodeling to predispose the altered ECM for tumor progression. Hence, understanding the means by which early Fn alterations occur during tumorigenesis may pave the way for the development of both diagnostic tools to halt cancer growth at early stages and therapeutics to prevent invasive cancer growth.

Tumor-associated Fn mechanobiology research is critical to deconvolute the diverse materials properties of the dysregulated tumor Fn, i.e., to distinguish among physical (matrix topology, molecular conformation), biochemical (binding affinity, sequestration), and biomechanical (elasticity, viscoelasticity) alterations during disease progression. For example, aging- and/or disease-induced Fn conformational changes occurring at the molecular scale (e.g., unfolding) dictate the binding of specific types of growth factors, integrins, and matrix components, which has deep implications in driving tumorigenesis. However, these molecular conformational changes are usually accompanied by concurrent topological and mechanical changes at a larger scale, which makes it difficult to unravel specific mechanisms and their chronology. As such, the recent advances made towards understanding the structure–function relationship of Fn in tumorigenesis highlight the importance of utilizing interdisciplinary approaches in cancer research.
